# The rat as a novel model for chronic rotator cuff injuries

**DOI:** 10.1038/s41598-024-55281-5

**Published:** 2024-03-04

**Authors:** Tao Yuan, Cheng-Teng Lai, Shao-Qiang Yang, Jia Meng, Hong Qian, Xin Yu, Hui Jiang, Qing-Gang Cao, Jian-Da Xu, Ni-Rong Bao

**Affiliations:** 1 Department of Orthopaedics, Nanjing Jinling Hospital, Affiliated Hospital of Medical School, Nanjing University, 305 Zhongshan East Road, Nanjing, 210002 China; 2 Department of Orthopaedics, Changzhou Traditional Chinese Medical Hospital, Changzhou hospital Affiliated to Nanjing University of Chinese Medicine, 25 North Heping Road, Changzhou, 213000 Jiangsu China

**Keywords:** Rat model, Chronic rotator cuff injuries, Behavior, Histologic, MRI, Inflammatory pain-related genes, Diseases, Geriatrics

## Abstract

Chronic rotator cuff injuries (CRCIs) still present a great challenge for orthopaedics surgeons. Many new therapeutic strategies are developed to facilitate repair and improve the healing process. However, there is no reliable animal model for chronic rotator cuff injury research. To present a new valuable rat model for future chronic rotator cuff injuries (CRCIs) repair studies, and describe the changes of CRCIs on the perspectives of histology, behavior and MRI. Sixty male Wistar rats were enrolled and underwent surgery of the left shoulder joint for persistent subacromial impingement. They were randomly divided into experimental group (n = 30, a 3D printed PEEK implant shuttled into the lower surface of the acromion) and sham operation group (n = 30, insert the same implant, but remove it immediately). Analyses of histology, behavior, MRI and inflammatory pain-related genes expression profiles were performed to evaluate the changes of CRCIs. After 2-weeks running, the rats in the experimental group exhibited compensatory gait patterns to protect the injured forelimb from loading after 2-weeks running. After 8-weeks running, the rats in the experimental group showed obvious CRCIs pathological changes: (1) acromion bone hyperplasia and thickening of the cortical bone; (2) supraspinatus muscle tendon of the humeral head: the bursal-side tendon was torn and layered with disordered structure, forming obvious gaps; the humeral-side tendon is partially broken, and has a neatly arranged collagen. Partial fat infiltration is found. The coronal T2-weighted images showed that abnormal tendon-to-bone junctions of the supraspinatus tendon. The signal intensity and continuity were destroyed with contracted tendon. At the nighttime, compared with the sham operation group, the expression level of IL-1β and COX-2 increased significantly (P = 0063, 0.0005) in the experimental group. The expression of COX-2 in experimental group is up-regulated about 1.5 times than that of daytime (P = 0.0011), but the expression of IL-1β, TNF-a, and NGF are all down-regulated (P = 0.0146, 0.0232, 0.0161). This novel rat model of chronic rotator cuff injuries has the similar characteristics with that of human shoulders. And it supplies a cost-effective, reliable animal model for advanced tissue engineered strategies and future therapeutic strategies.

## Introduction

Chronic rotator cuff injuries (CRCIs), a most common chronic degenerative musculoskeletal injury, still present a great challenge for orthopaedics surgeons^1^. It usually manifests with shoulder pain and dysfunction due to continuous loss of tendon elasticity and structure. The poor innate reparability of rotator cuff tendons is not sufficient for complete functional repair itself, followed by a high failure rate (between 13 and 94%) after surgical repair^2^.

It is generally accepted that healing is worse for chronic tears than acute tears, but the reasons are still unknown^3^. Many new therapeutic strategies (such as new surgical strategies, steroid injection, manual therapy, and platelet-rich plasma) are developed to facilitate repair and improve the healing process. However, prior to use in humans, it’s necessary to test these new strategies in animal models and computer simulation^4^.

The etiology of chronic tendon injury is multifactorial. So far, the tendon specimens of human studies are usually obtained from symptomatic patients. The early stages of the disease are still difficult to understand^5^. It’s essential to establish an animal model to improve understanding of pathophysiology of CRCIs and test new therapeutic strategies.

In contrast to other diseases, few robust animal models of tendon injury exist to sufficiently simulate all the features in human shoulders. The animal models must be established based on the specific research questions. Traditionally, larger animals are often used as model animals of rotator cuff injuries because of their anatomic similarity to that of humans^6^. However, small animal models are increasingly accepted recently. Compared with other different animal models, rat is regarded as a good candidate for rotator cuff’s pathology study. It has the similar anatomy to the rotator cuff of human with high cost-effectiveness^7,8^

Subacromial impingement, a risk factor causing rotator cuff injuries, is not fully understood so far^9^. To the best of our knowledge, there has been few reports concerning animal models of CRCIs with a persistent impingement. Thus, this study presented a novel reliable rat model of CRCIs that could mimic the degenerative alterations in rotator cuff for further study. A 3D printed PEEK implant was shuttled into the lower surface of the acromion to create persistent impingement. And we further aimed at confirming the functional impairment of forelimb is similar to human shoulder on the perspectives of histology, behavior, MRI and inflammatory pain-related genes expression profiles.

## Materials and methods

### Rat animal model

Sample sizes were established by power analysis. Sixty male Wistar rats (weighing 280 ± 20 g), housed in light- and temperature-controlled rooms with a standard diet were enrolled in this study. All enrolled rats were randomly divided into experimental group (n = 30) and sham operation group (n = 30), and fasted overnight before surgery.

### Design and fabrication of implants

**Figure 1:**
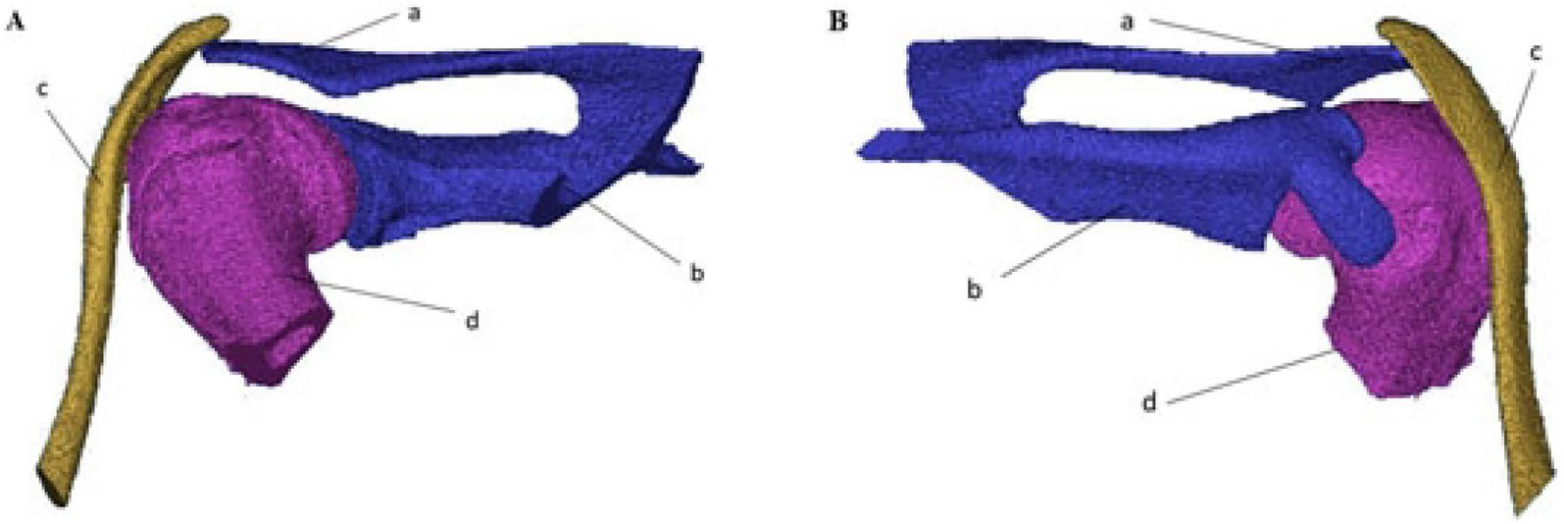
Three-dimensional reconstruction model of CT scan of shoulder in rat. (**A**) Rear view of left shoulder of rat, a: acromion b: scapula c: clavicle d: humerus; (**B**) front view of rat left shoulder, a: acromion b: scapula c: clavicle d: humerus.

**Figure 2:**
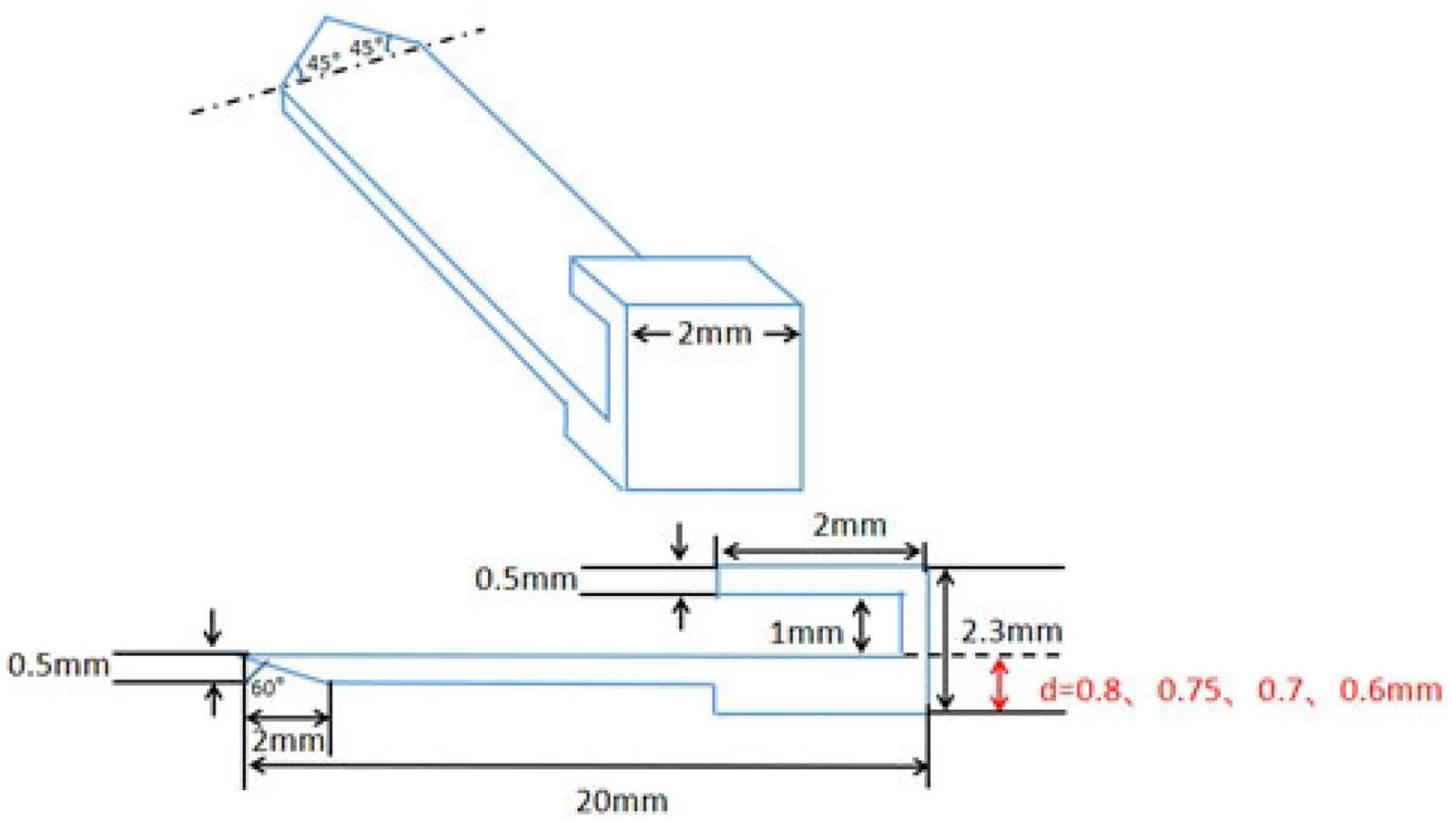
The structure of 3D printed PEEK implant.

The small animal CT machine was used to scan the shoulder joints of rats, and the implants were designed according to the three-dimensional reconstruction model (Fig. 1). The designed implant was printed by a medical PEEK 3D printer (P350, Pro 3D Tribal Shanghai Technology Co., Ltd.). The schematic diagram of the implant structure is shown below (Fig. 2).

### Surgical procedure

All rats were anesthetized by intraperitoneal injection with 2% sodium pentobarbital (40 mg/kg). After anesthesia, they were placed in prone position with the forelimbs in external rotation, and fur in the left shoulder joint was shaved as the surgical site.

### Experimental group

**Figure 3:**
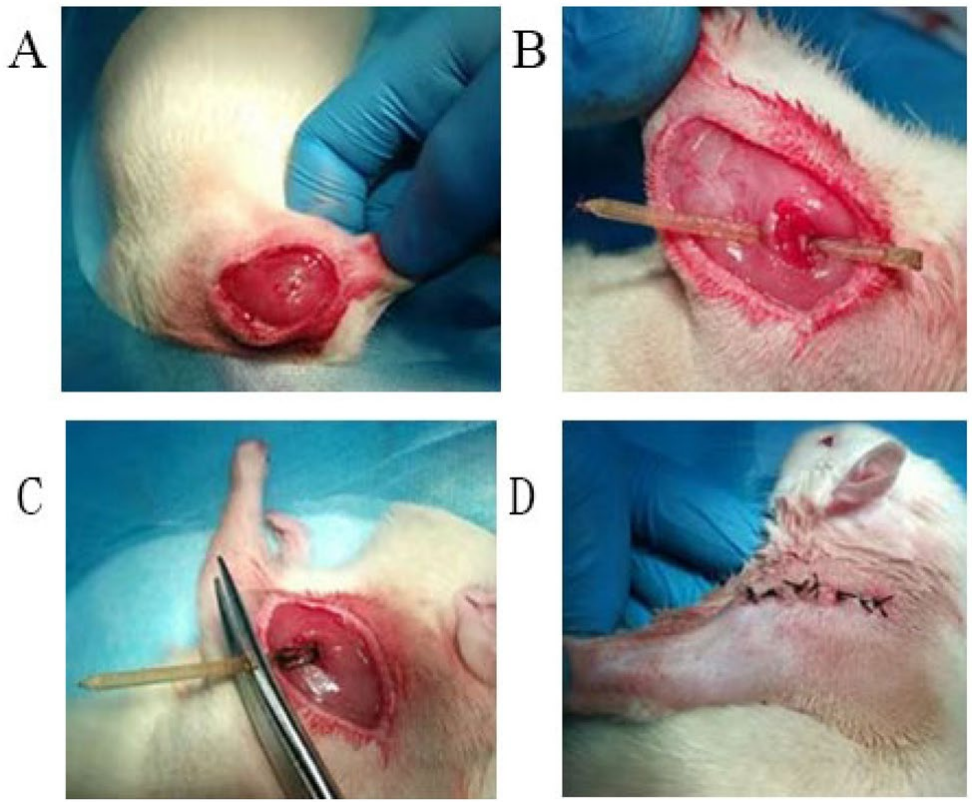
Surgical procedure of rat model. (**A**) Expose the acromion and deltoid muscle; (**B**) a 3D printed PEEK implant was shuttled; (**C**) the excess part of implant over the acromion is cut off; (**D**) the incision was then closed with a No. 2 silk thread.

A 2 cm skin incision parallel to the scapula was made under sterile conditions, followed by detachment of underlying fascia and the trapezius. A 5 mm incision along the lower edge of the trapezius and the scapula to expose the acromion and deltoid muscle. The acromion was lifted to help expose the area of the subacromial space for implant entry. A 3D printed PEEK implant was shuttled into the lower surface of the acromion to induce persistent sub-acromial impingement. To avoid lateral migration, the implant penetrated the posterior edge of the deltoid muscle from the posterolateral of the acromion. The implant was fixed with a No. 4 silk thread over the top of the acromion in a crossing fashion. And the excess part of implant over the acromion is cut off. The incision was then closed with a No. 2 silk thread and penicillin was injected intramuscularly to prevent infection (Fig. 3).

### Sham operation group

In the sham operation group, the same method was used to insert the same implant. After that, the implant was removed immediately.

The skin wound was sutured and Benzylpenicillin Sodium (1,600,000 unit/ml, Shandong Lukang Pharmaceutical Co., Ltd, China) was injected intramuscularly to prevent infection. The rats were resuscitated and recovered under heat lamps until they were transferred to cages. The rats had no serious lameness about 3 days after the operation. All the rats in the two groups started to run on the treadmill for 8 weeks^10,11^. Considering the rats had not fully adapted, the parameters of the small animal treadmill on the first 2 days could be set to: the inclination angle of the downhill 13°, the speed 15–17 m/min, and the time 25 min/day. From the third day of running, the parameters of the small animal treadmill can be set to: 13° downhill inclination angle, speed 17 m/min, time 30 min/day.

### Histological examination

After 2, 4 and 8 weeks of running on the treadmill, 6 rats from the experimental group and 6 rats from the sham operation group were selected respectively. All these rats were deeply anesthetized by intraperitoneal injection (2% sodium pentobarbital, 100 mg/kg) and killed. The shoulder joint specimens were harvested, and cut into a size of 6 mm × 6 mm × 6 mm. The bottom surface of the specimen was parallel to the direction of the supraspinatus tendon. They were placed in 4% paraformaldehyde for 24 h, dehydrated with gradient ethanol, and embedded in resin after tissue penetration. A Lycra hard tissue microtome with a tungsten steel blade was used for sectioning. The section thickness is about 4 μm. Haematoxylin and Eosin (HE) and Van Gieson staining were used to observe the pathological changes of the supraspinatus tendon under light microscope.

### Real-time PCR

Partial specimens for histological examination at daytime (9:00–10:00 a.m.) were obtained for real-time PCR. And another 6 rats from the experimental group and the sham operation group were killed after 12 h at the same day ((9:00–10:00 p.m.). All these tissues were stored in RNA later preservation solution for fluorescent quantitative PCR detection to compare the relative expression levels of the upstream and downstream genes, of which are related to inflammation in pain signaling pathways.

After the tissue was ground, RNA was extracted using the Trizol method RNA kit, and the RNA was extracted using a silica matrix adsorption column method. The HiScript 1st Strand cDNA Synthesis Kit was used for reverse transcription of the extracted RNA. Then use SYBR Green I method for quantitative real-time PCR using reagents: AceQ qPCR SYBR Green Master Mix (without ROX). The primer sequences used for gene detection are shown in Table 1. Here is the qPCR algorithm (relative quantification, 2-ΔΔCt method) formula:

**Table 1:** The primers information in this study.

Genes	Forward sequence (5ʹ–3ʹ)	Reverse sequence (5ʹ–3ʹ)	PCR products(bp)
GAPDH	TCTCTGCTCCTCCCTGTTC	ACACCGACCTTCACCATCT	87
NGF	AAGGCTTTGCCAAGGACG	CCTCTGGGACATTGCTATCTGT	141
IL-1β	CTATGGCAACTGTCCCTGAAC	GCTTGGAAGCAATCCTTAATCT	112
COX-2	TCAATGAGTACCGCAAACGC	TGGTCTCCCCAAAGATAGCA	177
TNF-a	GCCACCACGCTCTTCTGTC	GCTACGGGCTTGTCACTCG	149

$$ \begin{aligned}   {\text{RQ}} =  & \frac{{2^{{ - (Sample{\text{ Ct}}_{{{\text{targ}}}}  - Cont{\text{ Ct}}_{{{\text{targ}}}} )}} }}{{2^{{ - (Sample{\text{ Ct}}_{{{\text{ref}}}}  - Cont{\text{ Ct}}_{{{\text{ref}}}} )}} }} \\     =  & \frac{{2^{{ - (Sample{\text{ Ct}}_{{{\text{targ}}}}  - Sample{\text{ Ct}}_{{{\text{ref}}}} )}} }}{{2^{{ - (Cont{\text{ Ct}}_{{{\text{targ}}}}  - Cont{\text{ Ct}}_{{{\text{ref}}}} )}} }} \\     =  & 2^{{ - [({\text{SampleCt}}_{{{\text{targ}}}}  - {\text{ Sample Ct}}_{{{\text{ref}}}} {\text{) }} - {\text{ (Cont Ct}}_{{{\text{targ}}}} {\text{ }} - {\text{Cont Ct}}_{{{\text{ref}}}} )]}}  \\  \end{aligned}  $$.

### MRI evaluation

The remaining 6 rats in the experimental group and the sham operation group were deeply anesthetized and killed. The rats were evaluated by a 7.2 T MRI Biospect system (Bruker, Germany) in a supine position. The forelimbs were placed onto their abdomen. The shoulder was independently evaluated using Rapid Acquisition with Relaxation Enhancement (RARE) 1 mm axial TR 4000 T 30 sequence in axial and sagittal oblique planes parallel to the scapula. Osirix (Apple) was employed to analysis the continuity of rotator cuff.

### Statistical analysis

SPSS 24.0 statistical software was employed for analysis. The normality of distribution for continuous numeric variables was assessed by Kolmogorov–Smirnov test. According to normally distributed or not, the variables were presented as means with SD, and otherwise as medians with inter-quartile ranges (95% confidence intervals, 95% CI). In the histological and pathological analysis, Fisher’s exact test was used to compare the incidence of rotator cuff injury between groups. The statistical significance of the differences between groups was analyzed by one-way analysis of variance (ANOVA) to test the homogeneity of variance, and then the S–N–K (s) t test was performed. In all cases, P < 0.05 was considered statistically significant. The positive statistical significance was set at P < 0.05.

### Ethical approval

This study was carried out in compliance with the arrive guidelines (http://www.nc3rs.org.uk/page.asp?id=1357). All animals received humane care in compliance with "The Principles of Laboratory Animal Care" formulated by the National Society of Medical Research and "The Guide for the Care and Use of Laboratory Animals" published by the U.S. The animals were housed at a set temperature (24 °C) in a humidity-controlled room with a 12-h light/dark photoperiod. All experimental procedures were carried out strictly in accordance with the care and use of laboratory animals, which were approved by the National Institute of Health's Guide for the Care and Use of Laboratory Animals and the Institutional Animal Care and Use Committee at Nanjing University, and registered with the Ethics Committee of Jinling Hospital (2022DZGKJDWLS-0024). All animals were anesthetized with ether inhalation before operation.

## Results

**Figure 4:**
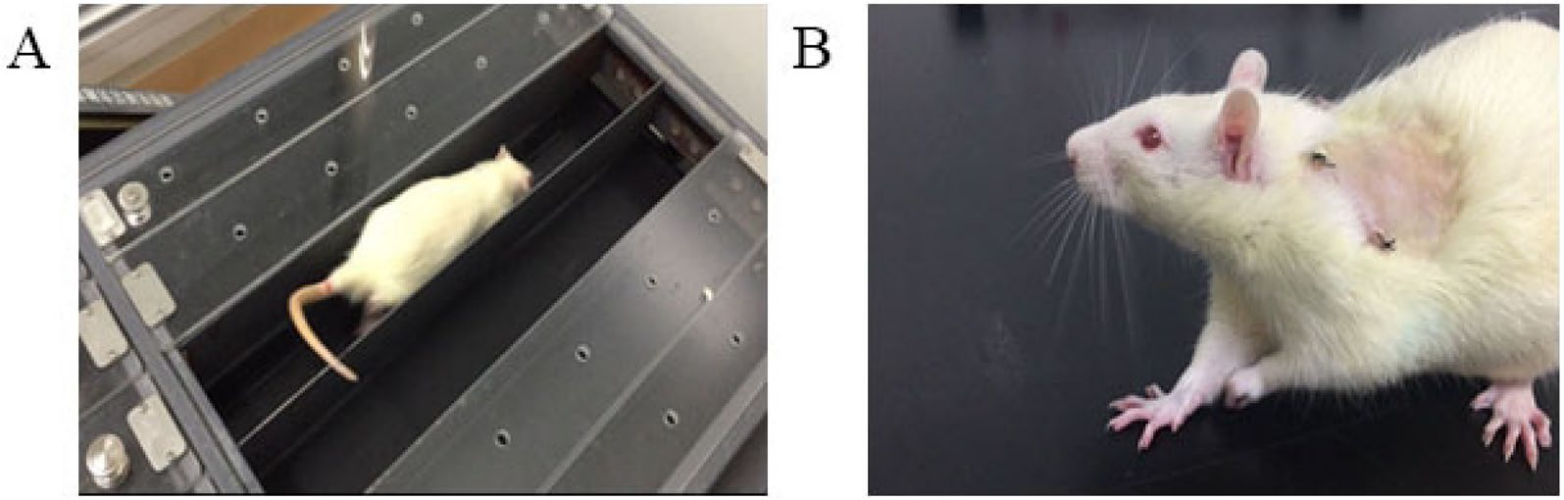
Experimental process and general performance of rats after 2-weeks running. (**A**) Rat is running on the treadmill; (**B**) in the experimental group, the rat’s forelimb on surgical side did not touch the ground after 2-weeks running.

All rats survived to the end of the experiment and had no infection. The rats had no serious lameness about 3 days after the operation and started to run on the treadmill. After about 2 weeks of running, the rats in the experimental group exhibited compensatory gait patterns to protect the injured forelimb from loading after 2-weeks running (Fig. 4).

### Histologic tendinopathic changes

**Figure 5:**
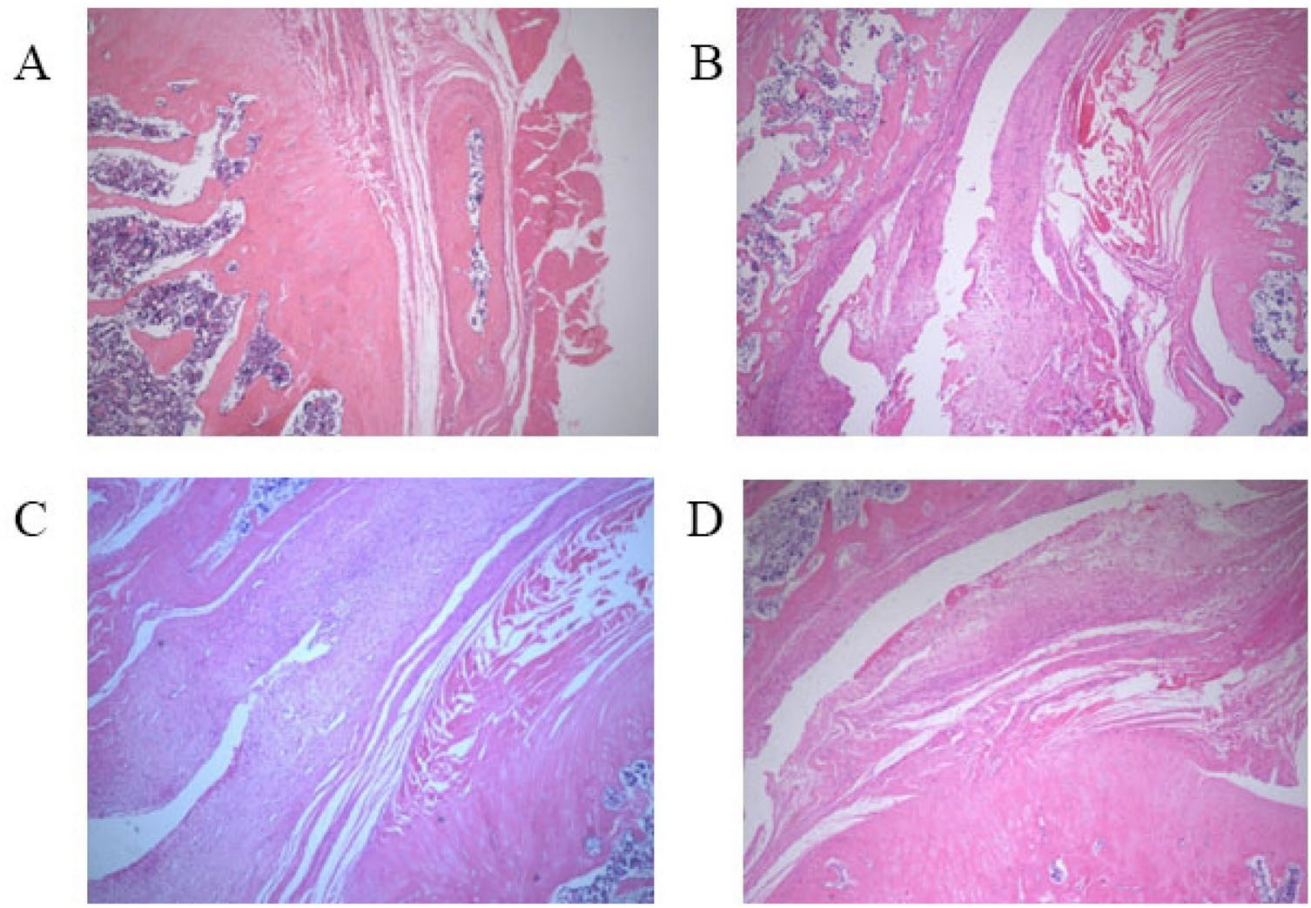
Histologic evaluation (H&E, ×40) of tendon degeneration. (**A**) Sham operation group: long, spindle shaped fibroblasts aligned between collagen fibers; (**B**) chronic model at 2 weeks: rough and irregular collagen fibers; (**C**) chronic model at 4 weeks: partial fat infiltration of tendon detachment; (**D**) chronic model at 8 weeks: poorly organized collagen fibers with fat interposition at the bursal-side tendon, while better collagen organization at humeral side.

**Figure 6:**
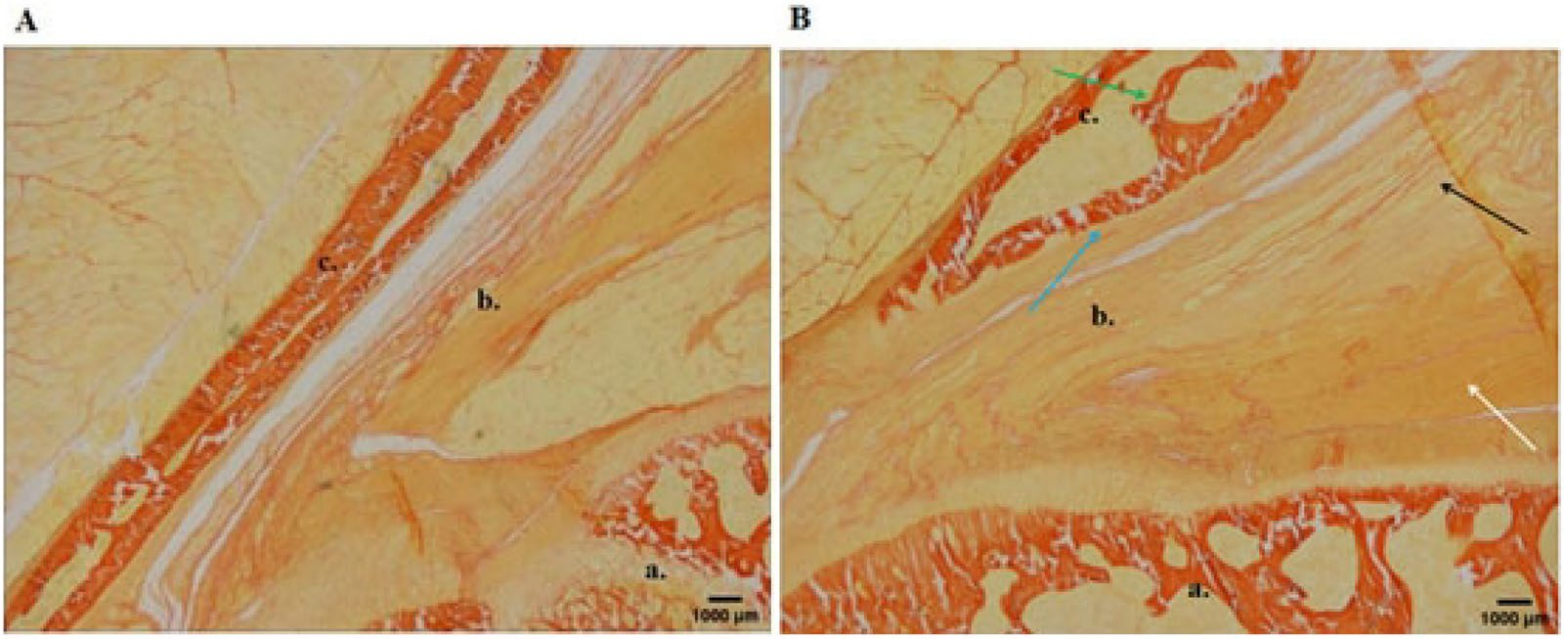
Van Gieson staining of the supraspinatus muscle tendon at the proximal end of the humeral head after 8 weeks (×40). (**A**) Sham operation group; (**B**) operation group (a. humerus head b. supraspinatus muscle tendon c. acromion; the green arrow indicates acromion bone hyperplasia; the blue arrow indicates acromion periosteal thickening; the black arrow indicates the bursal-side tendon was torn and layered, forming gaps; and the white arrow indicates part of the inter tendon on humeral side is neatly arranged collagen).

**Figure 7:**
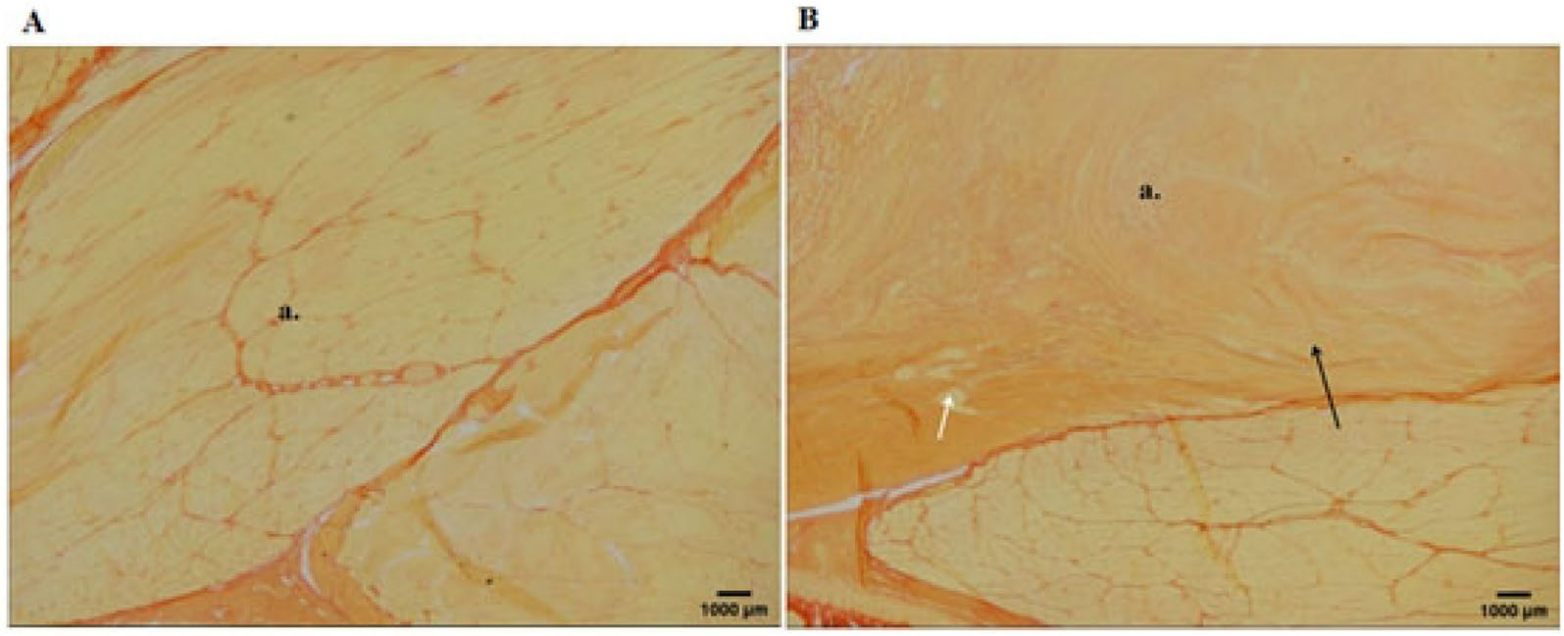
Van Gieson staining observation of the supraspinatus muscle tendon at the distal end of the humeral head after 8 weeks (×40). (**A**) Sham operation group; (**B**) operation group (a. supraspinatus muscle tendon; black arrow indicates internal tear of supraspinatus muscle tendon, structural disorder, obvious collagen degeneration of tendon; white arrow indicates partial broken tendon at humeral side).

After 8-weeks running, rats in the sham operation group had flat acromion, smooth supraspinatus tendon edges, no internal tearing and delamination of the tendon, clear structure, and no collagen degeneration. The rats in the experimental group showed obvious CRCIs pathological changes: (1) acromion bone hyperplasia and thickening of the cortical bone; (2) supraspinatus muscle tendon of the humeral head: the bursal-side tendon was torn and layered with disordered structure, forming obvious gaps; the humeral-side tendon is partially broken, and has a neatly arranged collagen. Partial fat infiltration is found (Figs. 5, 6, 7).

### Changes in inflammatory pain-related genes expression profiles

**Figure 8:**
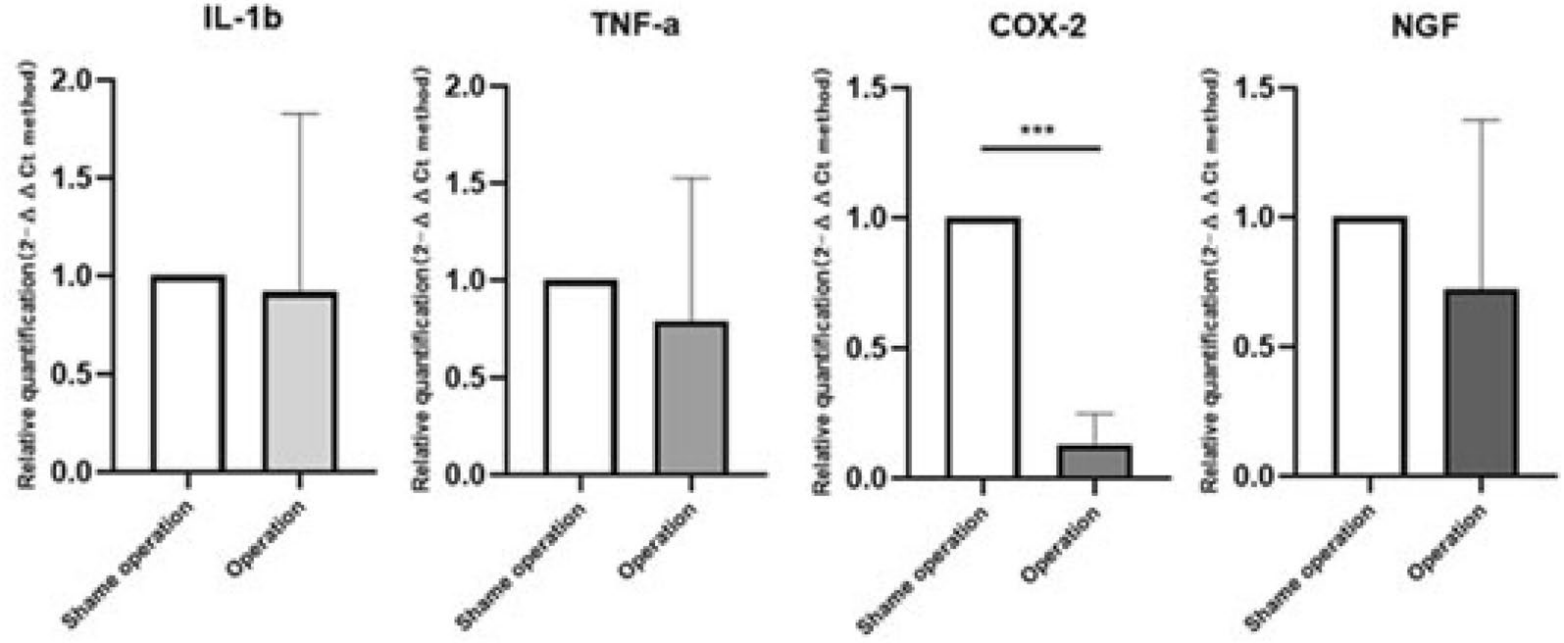
Comparison of inflammatory pain-related genes in the experimental group and the sham operation group at daytime (9:00–10:00, a.m.). Compared with the sham operation group, the expression of COX-2 in the experimental group was significantly lower (P = 0.0002). The expression levels of IL-1β, TNF-a, and NGF were all down-regulated compared with the sham operation group, but there was no significant difference (P > 0.05).

**Figure 9:**
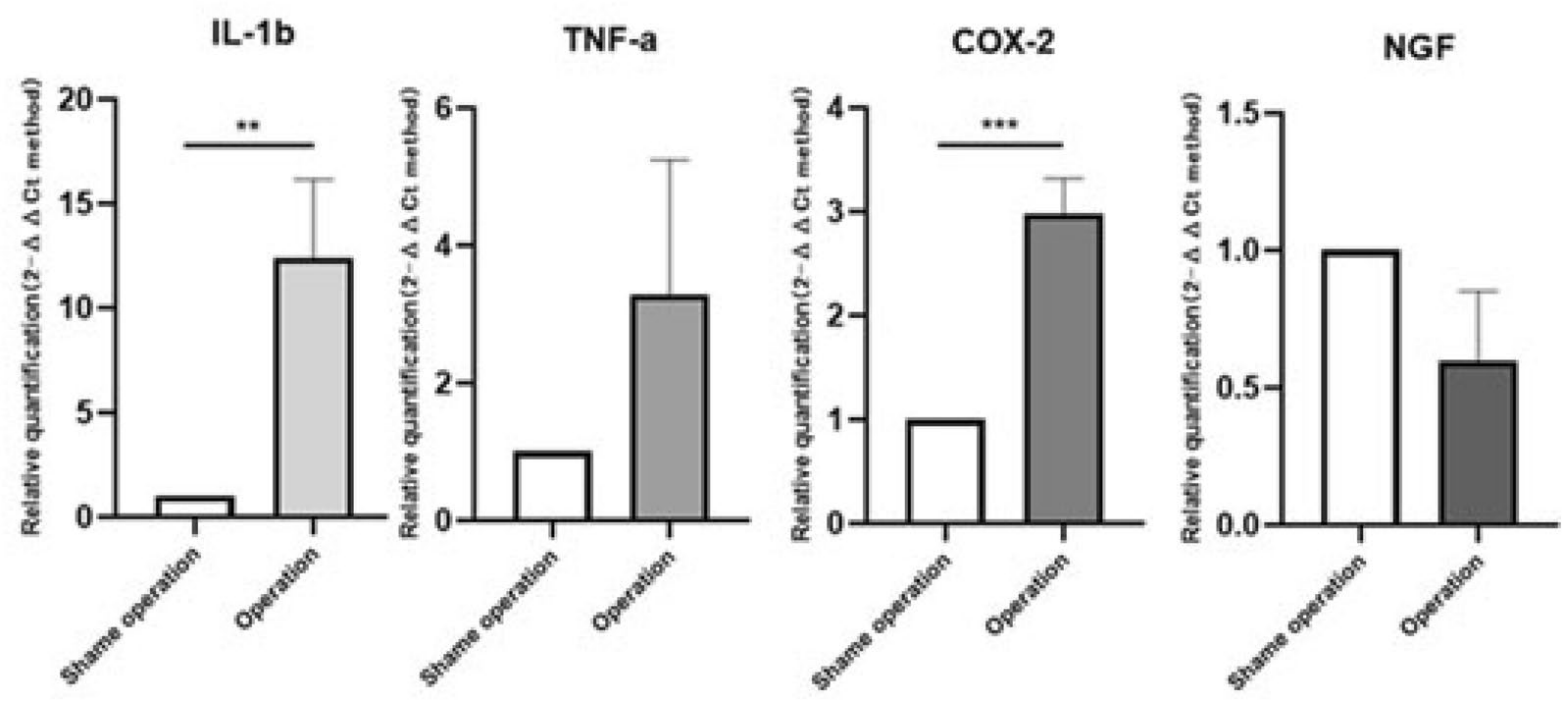
Comparison of inflammatory pain-related genes in the experimental group and the sham operation group at nighttime (9:00–10:00, p.m.). Compared with the sham operation group, the expression level of IL-1β and COX-2 increased significantly (P = 0063, 0.0005) in the experimental group. Although the expression of TNF-a in the experimental group was up-regulated, there was no significant difference compared with the sham operation group. In addition, the expression of NGF in the experimental group was down-regulated and there was no significant difference (P > 0.05).

At daytime, compared with the sham operation group, the expression of COX-2 in the experimental group was significantly lower (P = 0.0002, Fig. 8). The expression levels of IL-1β, TNF-a, and NGF were all down-regulated compared with the sham operation group, but there was no significant difference (P > 0.05, Fig. 8). However, at the nighttime, compared with the sham operation group, the expression level of IL-1β and COX-2 increased significantly (P = 0063, 0.0005, Fig. 9) in the experimental group. Although the expression of TNF-a in the experimental group was up-regulated, there was no significant difference. In addition, the expression of NGF in the experimental group was down-regulated and there was no significant difference (P > 0.05, Fig. 9).

**Figure 10:**
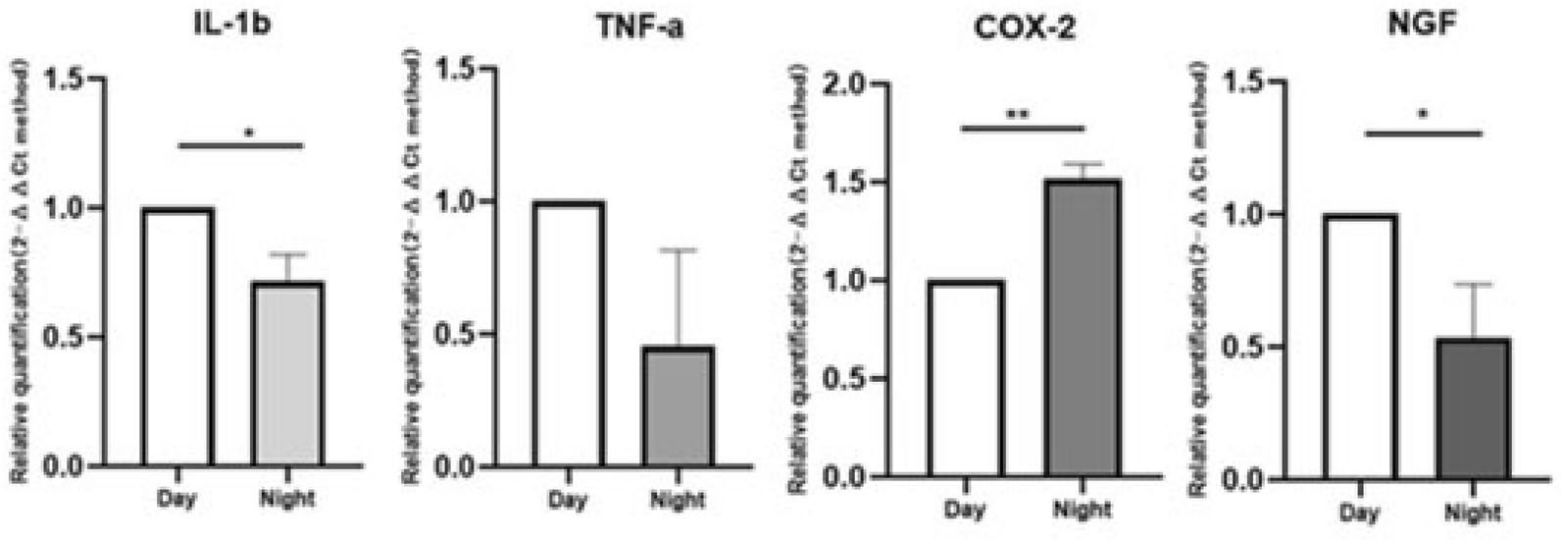
Inflammatory pain-related genes in the experimental group tested at daytime and nighttime. At nighttime, the expression of COX-2 is up-regulated about 1.5 times than that of daytime (P = 0.0011), the expression of IL-1β, TNF-a, and NGF are all down-regulated (P = 0.0146, 0.0232, 0.0161).

**Figure 11:**
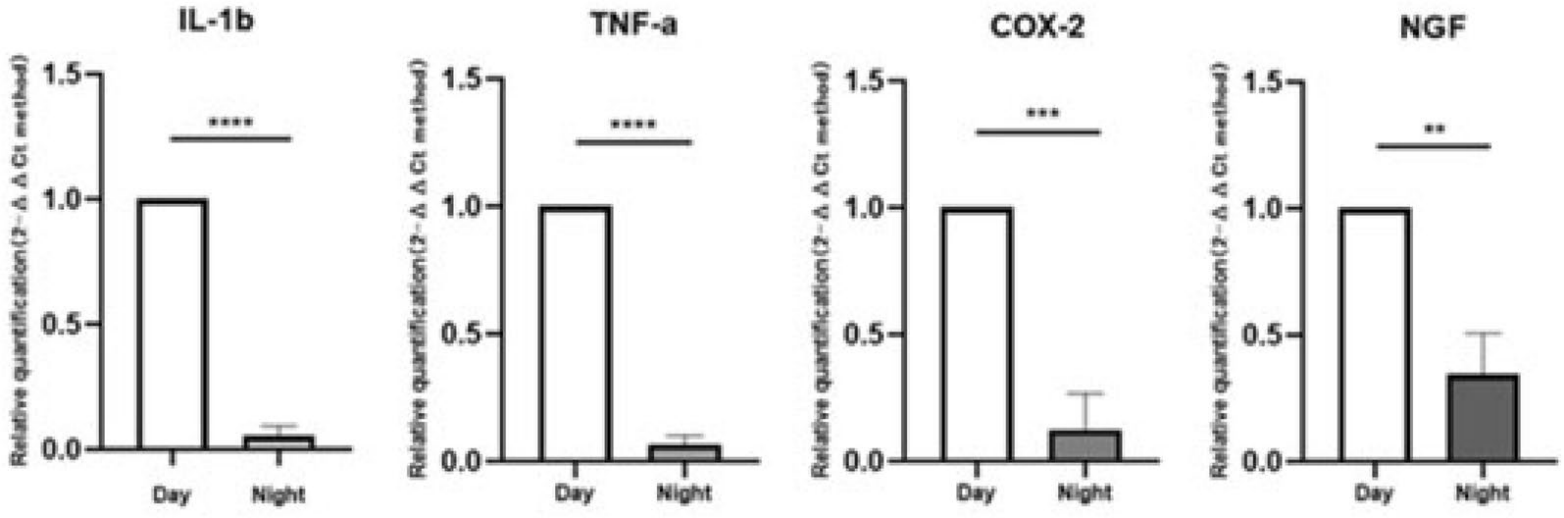
Inflammatory pain-related genes in the shame group tested at daytime and nighttime. At nighttime, IL-1β, TNF-a, COX-2, and NGF were significantly decreased (P ≤ 0.0001, < 0.0001, 0.0005, 0.0047, respectively). Compared with experimental group, the expression of COX-2 decreased significantly, but the expression of NGF was not significantly lower.

At nighttime, the expression of COX-2 in experimental group is up-regulated about 1.5 times than that of daytime (P = 0.0011, Fig. 10), but the expression of IL-1β, TNF-a, and NGF are all down-regulated (P = 0.0146, 0.0232, 0.0161, Fig. 10). However, all the expression of IL-1β, TNF-α, COX-2, and NGF in sham group significantly decreased (P ≤ 0.0001, < 0.0001, 0.0005, 0.0047, respectively, Fig. 11).

### MRI evaluation of affected supraspinatus muscle tendons

**Figure 12:**
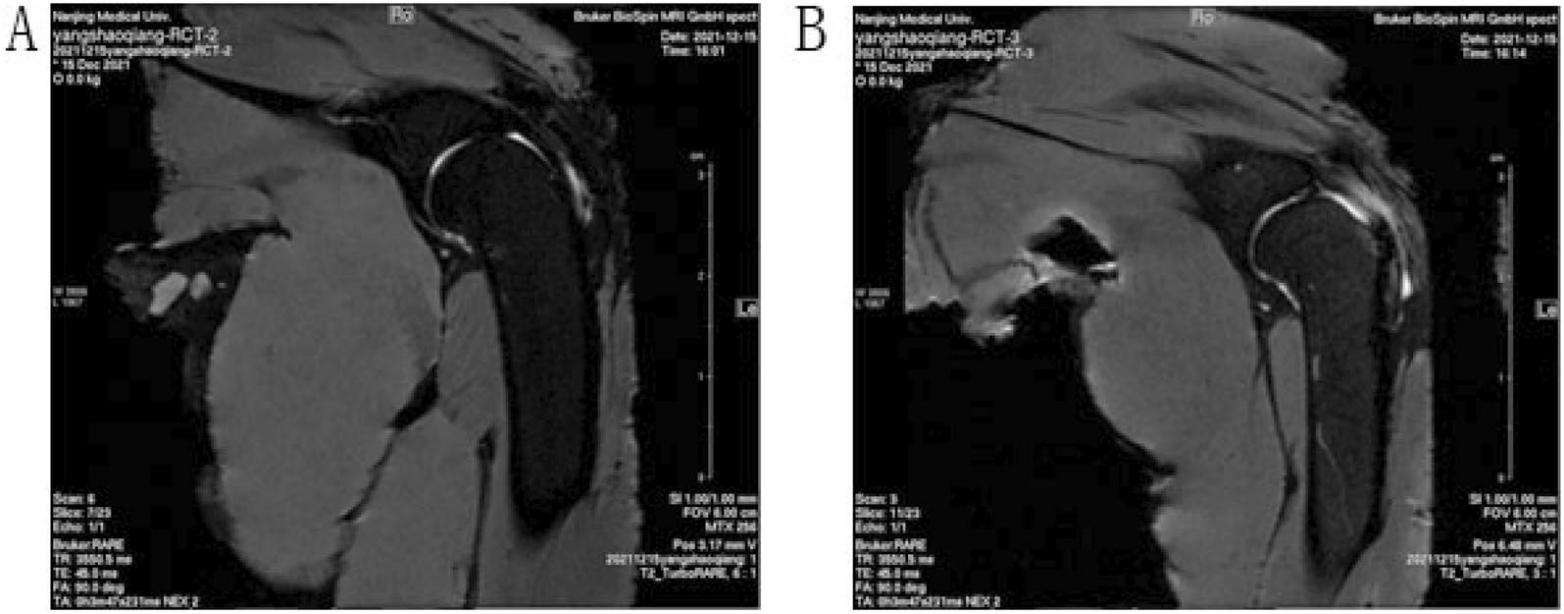
MRI evaluation of affected Supraspinatus muscle tendons. (**A**) Normal tendon-to-bone junctions in sham operation group; (**B**) the signal intensity and continuity were destroyed in the supraspinatus tendon.

The coronal T2-weighted images showed that abnormal tendon-to-bone junctions of the supraspinatus tendon. The signal intensity and continuity were destroyed with contracted tendon (Fig. 12).

## Discussions

In present study, we presented a more reliable rat model for future studies of CRCIs. The function of shoulder was impaired, similar to the clinical manifestations of human beings. And histological, MRI and inflammatory pain-related genes expression profiles results confirmed the exist of increased inflammatory pain-related genes expression, partial destroyed tissue integrity, atrophy and fatty degeneration of the supraspinatus muscle.

Currently, a cost-effective animal model that mimics the characteristics of injuries itself is urgently needed^12^. Small animals, particularly rat, are generally a better fit for research at the cellular and molecular levels. They can best meet the needs of modern biologic research tools. As the mainstay of biologic laboratory investigations, rats allow genetic manipulations to demonstrate biological mechanisms of tendon healing. Meanwhile, the rats have the similar rotator cuff function with human, including high functional loads in the tendon and a coracoacromial-arch-like structure^13^. Soslowsky et al. developed a 34-item checklist of criteria to search an appropriate animal model in 33 different animals. The anatomic relationship between supraspinatus tendon and the tissues above it in the subacromial region was regarded as a most important criterion. Of them, only one animal, the rat, had the similar anatomical location of acromion and the supraspinatus tendon with that in humans. The rat was the only one that met all checklist criteria listed with a prominent supraspinatus tendon inserting on the greater tuberosity under an enclosed arch (the coracoid, acromion, and coracoacromial ligament). The changes in the supraspinatus tendon may have the similarities to human tendon disease^8^.

Persisting defect is a typical feature for human chronic tendon injuries. However, the rat has an early healing ability, which is a major challenge to establish a chronic model. In fact, subacromial impingement is an important mechanism contributing to CRCIs. The stenosis of the subacromial space, resulted from abnormal acromial morphology and the thickened coraco-acromial arch, causes inflammation and persistent physical damage to the muscle and tendon passing through the subacromial space^14^. Accumulation of repetitive microtrauma eventually results in chronic rotator cuff injuries, even full-thickness tears. To sufficiently simulate all the features in human shoulders, a rat animal model of subacromial impingement was developed by wrapping allograft Achilles tendon around the acromioclavicular arch. And the tendon tissue architecture changed with increased fibroblast accumulation and decreased collagen^8^. However, in contrast to soft tissue compression, our model simulates bony impingement with a 3D printed PEEK implant shuttled into the lower surface of the acromion. The mechanical wear at the PEEK-tendon interface avoids the direct damage of chemical or surgical means, and prevents self-healing and reattachment of the cuff defects. The accumulation of microscopic matrix damage ultimately weakens rotator cuff tendon after repetitive tendon loading (tendon overuse and impingement)^15^. Our study confirmed the appearance of decreased biomechanical tissue integrity after 8-weeks running. The bursal-side tendon of proximal supraspinatus muscle tendon was torn and layered, forming obvious gaps. And about 1/3 of the humeral-side tendon showing neatly arranged collagen. The surgical protocol of our modeling process was simplified and easily performed. Although no comparison is conducted between these surgical methods, we generally believe that rigid body-induced impingement can better simulate the rotator cuff injury. Further study is necessary to confirm this conclusion.

Overuse of supraspinatus tendon also plays an important role in CRCIs. The detachment of supraspinatus alone usually brings a high rate of tendinous adherences^13^. To emulate this mechanism, the overuse was induced through downhill running with an implant inserted into the lower surface of the acromion. In this study, we chose the overuse-induced rat model of CRCIs, because daily downhill treadmill running had the similar histological and mechanical alternations with that of human^16^. A constant damage was applied to the impinged tendon when the rats underwent intensive treadmill running. It hinders the healing of mouse tendons by creating a consistent milieu. This process is believed to better mimic the clinical situation of rotator cuff injury^17^. The damage and tears often occur in the insertion region of the tendon due to the complex local mechanical forces. The insertion region demonstrated flattened crimps and a loss of the periodicity of crimping due to exercise-induced microtrauma. The disrupted collagen alters the biomechanical properties^18^.

An ideal animal model usually needs to accurately simulate anatomical structures and pathological mechanisms as accurately as possible. From another perspective, the effectiveness of the model must be evaluated on the perspectives of histology and behavior^12^. The mechanical load on the forelimb of rat is different from that of human shoulder due to the human orthostatic position. However, gait analysis provides an objective indicator of successful animal models^19^. Thus, behavioral and functional analyses are needed during reconstruction of animal models^20^. In present study, the gait pattern was changed. The rats in the experimental group exhibited compensatory gait patterns to protect the injured forelimb from loading after 2-weeks running.

The degenerative changes were found in operated forelimb. Mechanical properties of rotator cuff decreased significantly with collagen disorganization after tendon detachment. A qualitative increase was found in fibroblast-like cells and vascularity^13^. Histopathology of full-thickness supraspinatus tendon biopsy specimens in human showed increased cellularity, collagen disorganization, and rounded cell nuclei^21^. Other studies also had the similar conclusions. There was a decrease in collagen organization and increased cellularity with more rounded cell shape after chronic rotator cuff tears^22^. After insertion of a 3D printed PEEK implant, we observed histological changes consistent with chronic tendon degeneration in the supraspinatus tendon. These changes including decreased organization, increased cellularity, and rounded cell shape.

Time-post-tear affects the effect of surgical treatment. The disorganization of collagen fibers increased from 4 weeks after surgery and lasted to 16 weeks^22^. After detachment of the rat supraspinatus tendon, the changes of degeneration and loss of muscle mass recovered after 9 weeks. The histological changes between 6 and 9 weeks were similar with that of the human tendon^23^. Histological analysis had confirmed that structural damage of rotator cuff occurred after 8-weeks downhill running^24^. We also found that obvious changes to the supraspinatus tendon occurred after 8-weeks running. The tendon manifested with fiber separation from bundles and increased cell rounding.

Our study demonstrated that the supraspinatus changed significantly, including atrophy and fat accumulation. Previous study concluded that the suprascapular nerve section could improve fatty infiltration and atrophy. However, we chosen avoidance of procedure. The neurotmesis did not faithfully represent the degenerative alterations in chronic rotator cuff tear^25^. Fatty infiltration and accumulation of fibrotic tissue are reactions of muscle to chronic rotator cuff injuries in human. Fatty infiltration and muscle atrophy are irreversible, which are associated with poor prognosis after arthroscopic surgery^26^. However, the pathologic process of fatty infiltration and atrophy have not been fully elucidated. In the rat models of chronic rotator cuff tear, there are contrary reports about degenerative changes. Barton et al. reported that no significance in fatty degeneration was found between groups at any time interval. The atrophy peaked at 4 weeks and recovered in the following weeks^27^. Hashimoto et al. reported that the rat rotator cuff tear model had accurate chronic histologic changes till 12 weeks after surgery. Muscle atrophy improved at 12 weeks after surgery, but fatty infiltration did not decrease even after the defect healed^28^. Fatty infiltration could not be considered as a determinant on the time interval for the rat model. Whereas another group reported that there were significant changes including fatty infiltration, a loss of muscle mass and loss of normal collagen fiber structure/arrangement 3 weeks after detachment of supraspinatus^29^. Thomazeau et al. reported that the supraspinatus muscle volume decreased in patients with chronic rotator cuff tears, but recovered with time at least half a year^30^. The fatty infiltration was found about 6 weeks after surgery in a rat model of massive rotator cuff tears^31^. Currently, it's still difficult to examine the fatty infiltration and atrophy into the rotator cuff due to unable to establish a model of significant fat accumulation.

The etiopathogenesis of CRCIs remains unclear, but inflammation plays a significant role in each stage of disease progression (initiation, progress and resolution)^32^. Previous study also confirmed that inflammatory markers played a role in impingement-related tendon damage^9^. The elevated levels of inflammatory mediators contribute to the shoulder pain in patients with rotator cuff injuries^33^. Pro-inflammatory factors can up-regulate the expression of inflammatory and down regulate the expression of type I collagen in tendon, which will reduce the tensile strength and elastic modulus^34^. Frich LH et al. evaluated inflammatory and degenerative markers of 22 patients with full- thickness supraspinatus tendon tear and found that the expression of IL-1β and TNF-α increased. The inflammation usually combined with fatty infiltration and degeneration^35^.

Inflammatory changes may be aggravated with the change of day and night. Gene expression analysis of affected tendons usually can confirm tissue turnover during disease progression earlier. Aberrant expression levels of inflammatory pain-related genes were found in this study. In this study, compared with the sham operation group, the expression level of IL-1β and COX-2 at night increased significantly in the experimental group. The expression of COX-2 in experimental group is up-regulated about 1.5 times than that of daytime, but the expression of IL-1β, TNF-a, and NGF are all down-regulated. Perry et al. conducted a study to detective inflammatory and angiogenic markers in a rat model with supraspinatus tendon overuse injury. They reported that the levels of cyclooxygenase-2 and vascular endothelial growth factor increased from 1 day through 16 weeks of treadmill running. The maximum gene expression level of COX-2 occurred at 8 weeks of overuse. And it provided theoretical basis for the use of COX-2 inhibitors and other nonsteroidal anti-inflammatory drugs^36^.

Although inflammatory response is necessary for tendon healing, a dramatic high level may hinder or impaired tendon healing. The tissue repair ability differed at different time points, even opposite effects. Manning CN et al. reported that the inflammatory phase of healing should be considered about tendon healing. A greater than 4000-fold up-regulation of IL-1β was confirmed by Quantitative real-time reverse transcription-polymerase chain reaction (qRT-PCR)^35^. Modulation of the early inflammatory phase maybe a potential strategy for improving tendon repair. Gulotta et al. reported that inhibition of TNF-α improved the tendon healing with a better overall strength^33^. Wang et al. found that enhanced M2 macrophage polarization could improve the prognosis of chronic rotator cuff tendinopathy and proposed modulating the M1/M2 macrophage activity balance as a new target of treatment^37^. Nerve growth factor (NGF) is released from peripheral tissues after injurious stimulation^38^. It continuously elevated up to 56 days in rotator cuff tear, which was associated with severe shoulder pain. TNF-α and IL-1β could regulated the expression of NGF^39^. However, this study has reached completely opposite conclusions. The expression NGF was all down-regulated in both groups. Further research may be needed to determine the exact duration of NGF changes.

### Limitations

There were some important limitations to be identified in this present study: (1) the small enrolled sample size limits the power of analyses; (2) quantitative evaluation of muscle atrophy and fatty infiltration are necessary in future study; (3) the time points of analysis are just 9:00–10:00, a.m. and 9:00–10:00, p.m., it’s important to evaluate the dynamic changes in more time points; (4) another limitation is about the inflammatory pain-related genes expression profiles. Nocturnal pain is a common issue for patients with shoulder disorders. We are unable to determine the specific genes expression profiles responsible for nocturnal pain. Therefore, we hesitate to make any other conclusions except increased inflammatory reaction.

## Conclusion

The rat model described in this study represents the similar degenerative alterations of that occurring clinically on the perspectives of histology, behavior and MRI. And it supplies a cost-effective, reliable animal model for advanced tissue engineered strategies and future therapeutic strategies. Further studies about exact biological and biomechanical mechanisms of rotator cuff injury are needed.

## Data Availability

All data generated or analyzed during this study are included in this published article and are available from the corresponding author on reasonable request.
